# Membrane vesicle heterogeneity of *Streptococcus mutans* is associated with strain-specific cariogenicity

**DOI:** 10.1080/20002297.2026.2761687

**Published:** 2026-11-12

**Authors:** Han Wang, Wen Zhou, Yousheng Zeng, Yazhuo Li, Yuze Yang, Yu Zeng, Guowu Gan, Shuai Chen, Xiaojing Huang

**Affiliations:** a Clinical Research Center for Oral Tissue Deficiency Diseases of Fujian Province & Fujian Key Laboratory of Oral Diseases, Fujian Provincial Engineering Research Center of Oral Biomaterial, School and Hospital of Stomatology, Fujian Medical University, Fuzhou, China; b Stomatological Key Laboratory of Fujian College and University & Institute of oral homeostasis & Research Center of Oral Tissue Engineering, School and Hospital of Stomatology, Fujian Medical University, Fuzhou, China

**Keywords:** Dental caries, *Streptococcus mutans*, membrane vesicles, heterogeneity, cariogenicity

## Abstract

**Background:**

Streptococcus mutans (*S. mutans*) is a major etiological agent of dental caries, and pronounced heterogeneity in cariogenic phenotypes exists among different strains. The membrane vesicles (MVs) of *S. mutans* participate in biofilm formation, and their yield and cargo composition vary with environmental conditions. The relationship between MVs and strain-specific differences in cariogenicity remains unclear.

**Materials and methods:**

The cariogenic potential of the strains was validated using a rat caries model, and strain-specific cariogenic phenotypes were correlated with MV abundance, physicochemical properties, biological functions, and proteomic composition.

**Results:**

Compared with MVs from weakly cariogenic strains, those derived from highly cariogenic strains were larger in size and greater in quantity. These MVs reversed defects in biofilm formation, extracellular matrix synthesis, and acid production in UA159 Δ*gtfBC*. Increased exopolysaccharide synthesis and eDNA accumulation strengthened the biofilm matrix architecture and upregulated the transcription of the acidogenesis-related gene *ldh* in recipient bacteria. Proteomic analyses revealed that MVs from highly cariogenic strains exhibited distinct protein cargo profiles enriched in multiple virulence-associated proteins, including Gtfs.

**Conclusion:**

MVs derived from *S. mutans* strains display clear strain‑dependent heterogeneity. The differences in physicochemical properties, secretion abundance, biological functions, and protein cargo are associated with cariogenic potential.

## Key messages

- **Key Message 1**: Highly cariogenic strains release MVs in greater numbers and with larger diameters that are enriched in multiple virulence proteins.
- **Key Message 2**: MVs carrying specific virulence proteins promote the production of biofilm exopolysaccharides and the accumulation of eDNA, while altering the transcription of recipient bacteria, thereby facilitating the establishment of an acidic microenvironment within the biofilm.
- **Key Message 3**: This study clarifies that MVs derived from strains with distinct cariogenic potential are heterogeneous. These findings lay a theoretical foundation for the study of MVs and provide a deeper understanding of the pathogenic mechanism of *Streptococcus mutans*.

## Introduction

Dental caries are among the most common chronic oral conditions worldwide and have a harmful effect on oral health status and quality of life [1,2]. More than 2.5 billion individuals have untreated dental caries affecting permanent teeth, and more than 500 million children have untreated dental caries affecting primary teeth [3]. Consequently, this disease persists as a major global public health burden.

*Streptococcus mutans* (*S. mutans*) plays a key role in cariogenic biofilm formation because of its capacity to produce an extracellular matrix, generate acids, and tolerate acidic conditions [4–6]. In the presence of sucrose within the dental plaque microenvironment, *S. mutans* expresses and secretes glucosyltransferases (GTFs), which consist of three isoenzymes: GtfB, GtfC and GtfD. These enzymes utilize sucrose as a substrate to catalyze the biosynthesis of extracellular glucans. GtfB predominantly produces water-insoluble glucans enriched in *α*-1,3 glycosidic linkages, whereas GtfD mainly synthesizes water-soluble glucans enriched in *α*-1,6 glycosidic linkages, and GtfC contributes to the synthesis of both glucan fractions [7]. In particular, water-insoluble glucans serve as predominant structural constituents of the biofilm matrix. These molecules not only promote bacterial adhesion and microcolony formation but also help maintain the mechanical integrity of mature biofilms [8,9]. Extracellular DNA (eDNA) is likewise an indispensable structural element of biofilm matrices. Interactions between eDNA, bacterial cells and secreted glucan polymers strengthen matrix cohesion and preserve biofilm structural integrity [10–12]. Concurrent with matrix assembly, carbohydrate fermentation by plaque bacteria produces organic acids and drives local pH reduction. Acid‑tolerance systems of *S. mutans* support bacterial persistence and continued metabolic function under these acidic microenvironmental conditions. Collectively, extracellular glucan synthesis, eDNA-mediated matrix stabilization, acid production, and acid tolerance promote the development of structurally stable and persistently acidic cariogenic biofilms, ultimately contributing to the demineralization of hard dental tissues [6,13].

Core cariogenic pathways tend to be conserved across *S. mutans* populations, yet clinical isolates show considerable variability in their ability to drive caries development [14,15]. Genomic polymorphisms and variable expression of known virulence‑related genes account for part of this phenotypic heterogeneity [16–18]. Nevertheless, a clear mechanistic picture linking these molecular discrepancies to the pronounced phenotypic variation observed among strains is lacking. The identification of novel factors that underlie strain‑specific cariogenicity is needed to improve the understanding of the mechanisms underlying caries and enable more precise preventive and therapeutic approaches.

Over the past two decades, extracellular membrane vesicles (MVs) have been recognized as a broadly conserved bacterial strategy supporting secretion and pathogenic activities [19,20]. In Gram‑positive organisms, including *S. mutans*, MV biogenesis begins with focal outward bulging of the cytoplasmic membrane. To be released, these vesicles must cross the cell wall, which occurs at sites with locally remodeled peptidoglycan layers [12,21]. These nanoscale lipid-bilayer vesicles selectively package a wide range of bioactive cargo, including proteins, lipids, nucleic acids, and virulence-associated factors, and protect their cargo from extracellular degradation and facilitate its delivery to target sites [19,22]. Accordingly, MVs participate in diverse biological processes, including nutrient storage and transport [22], intercellular communication [23], and biofilm formation [24].

Research on *S. mutans*-derived MVs has only recently been reported. Previous studies have demonstrated that these vesicles carry parent-derived virulence components, including GTFs and eDNA [25–27], and can also modulate biofilm development in *S. mutans* and other oral microorganisms [12,25,28–30]. These findings suggest that MVs may contribute to cariogenic development. Strain-specific differences in cariogenic potential may be closely associated with variations in MV production or secretion [12,31]. Our previous study reported that strains with strong cariogenicity in vitro release more MVs with larger diameters [32]. However, the relationships between the characteristics, functions and protein composition of MVs and the cariogenicity of strains have not been fully elucidated.

On the basis of these observations, we hypothesized that interstrain differences in *S. mutans* cariogenicity are associated with MV heterogeneity. To test this hypothesis, we combined an in vivo animal model of dental caries with in vitro MV characterization, functional rescue experiments, and quantitative proteomic analysis. This study expands the current knowledge of *S. mutans* MVs by revealing strain-dependent heterogeneity in their properties and function and highlights this heterogeneity as a factor for further investigation in the context of strain-specific cariogenicity.

## Materials and methods

### Bacterial strains and media

Detailed information on the bacterial strains is provided in Table S1. The reference strains *S. mutans* UA159 and UA159 Δ*gtfBC* [33] (kindly supplied by the State Key Laboratory of Oral Diseases, Sichuan University) were used in this study. The oral clinical strains *S. mutans* 593 and *S. mutans* 18 have been previously described by our group [34,35]. Briefly, *S. mutans* 593 was isolated from a caries-active adult with multiple active carious lesions (decayed, missing, and filled teeth [DMFT] = 10, with no missing teeth and three nonrestored cavities), whereas *S. mutans* 18 was isolated from a caries-free adult (DMFT = 0). Both strains have been deposited in the Oral Microbiome Bank of China (OMBC) under accession numbers COCC24593 (*S. mutans* 593) and COCC24180 (*S. mutans* 18) [36]. The BioSample data for strains 18 and 593 are available under accession numbers SAMN63684660 and SAMN63686341, respectively, in the NCBI BioSample database. All strains were routinely cultured in Brain Heart Infusion (BHI) broth (Oxoid, UK). Sucrose (Biosharp, China) was added to the medium at a final concentration of 1% (w/v) for relevant experiments (BHIS). The samples were incubated at 37 °C in an anaerobic chamber supplied with mixed gas (80% N_2_, 10% CO_2_, and 10% H_2_).

### Phylogenetic analysis and gram staining

We performed phylogenetic reconstruction according to published protocols [37]. For the 16S rRNA gene-based phylogeny, 16S rRNA sequences were retrieved from NCBI GenBank and our assembled genomes. The sequences were aligned using ssu-align-0.1.1 with structure-guided alignment for the SSU rRNA. A neighbor-joining tree was constructed using MEGA 12. Bootstrap analysis with 1,000 replicates was performed to evaluate the branch support across the phylogenetic tree. The construction was performed on the Majorbio Cloud Platform (https://v.majorbio.com/).

Gram staining was performed using a commercial kit (Leagene Biotech, China). Briefly, logarithmic-phase bacterial smears were heat fixed, stained with crystal violet, treated with iodine as a mordant, decolorized with 95% ethanol for 20 s, and counterstained with safranin. Slides were rinsed and air-dried prior to observation. Bacterial cell morphology was visualized by light microscopy with a 100 × oil‑immersion objective lens.

### Growth curves

Bacterial growth was monitored using a Bioscreen plate reader (Bioscreen C Lab systems) as previously described [38]. Starting from logarithmic-phase cultures, the inoculum was adjusted to an OD_600_ of 0.2 and further diluted 10-fold in BHI broth, with uninoculated BHI broth serving as the blank control. Cultures incubated at 37 °C were monitored for 24 h. Biolink software (Lab systems) was used to determine the OD_600_ values every 30 min, with 5 s of shaking implemented before each measurement. Growth curves were plotted on the basis of data from three independent biological replicates using GraphPad Prism 9 (GraphPad Software, USA).

### Dental caries animal model

This animal study received approval from the Institutional Animal Care and Use Committee of Fujian Medical University (IACUC FJMU 2026-0215). Our animal‑model setup used the experimental schemes reported in a previous study [6]. Female SPF‑SD rats at 3 weeks of age were selected for model construction. To modulate the gut microbiota during the early induction period (days 1–3), streptomycin was incorporated into the drinking water. The rats were then maintained on cariogenic Diet 2000# (Shuyu Bio, China) alongside 5% (w/v) sucrose-supplemented drinking water until the experimental endpoint. From day 4 to day 7, the rats were orally inoculated with different *S. mutans* strains. Sterile phosphate-buffered saline (PBS) was used as the negative control. Animals were randomized into four groups: UA159, *S. mutans* 18, *S. mutans* 593, and negative control groups, with five rats per group. A *post hoc* power analysis was performed using G*Power (v3.1) based on a one-way ANOVA design (*α* = 0.05, total *n* = 20, four groups). The results indicated that the statistical power (1 − *β*) exceeded 0.99, confirming that the sample size provided sufficient power to detect treatment effects [39]. Cells were harvested by centrifugation at 4,000 × *g* for 5 min, washed twice with PBS, and resuspended in PBS to an OD_600_ of 0.5 (approximately 7 × 10^7^ CFU/mL for each strain; Fig. S1D). Oral swabs were used to deliver bacterial suspensions to the mandibular molars of test‑group animals, while PBS was applied as a negative control. Food and water were withheld from the rats for 30 min after each inoculation step. On day 8, saliva and tooth-surface-adherent bacteria were collected using oral swabs and plated on mitis-salivarius-bacitracin agar (MSBA; Topbio, China) containing 1% potassium tellurite solution. After a 48-hour incubation period at 37 °C in 5% CO_2_, colony growth was used to validate successful in‑vivo colonization. Throughout the modeling period, body weights were recorded every 5 days. On day 36, CO_2_ inhalation was used for animal euthanasia, and skull specimens were harvested for further analysis.

### Viable biomass assay and micro-CT

The dissected mandibles were sonicated in sterile PBS. The bacterial suspension harvested from each mandible was serially diluted 10-fold with sterile PBS. A 100-μL aliquot from each dilution was spread onto MSBA plates for selective enumeration of *S. mutans*. The plates were incubated for 48 h at 37 °C under the same anaerobic conditions as described above. After incubation, the number of colony-forming units (CFUs) was determined, and the CFU count per mandible was calculated based on the dilution factor and the number of colonies.

Following sonication and collection of the bacterial suspension, the mandibles were fixed in 4% paraformaldehyde (PFA) and subsequently imaged using microcomputed tomography (micro‑CT) on a NEMO® Micro CT system (PINGSENG, China). Scanning parameters were set in accordance with the standard protocol for large ex vivo specimens at medium resolution. Imaging was performed in continuous scan mode, with a tube voltage of 90 kV, a tube current of 0.04 mA, and FDK reconstruction, yielding an isotropic voxel size of 0.03 × 0.03 × 0.03 mm^3^. All the scanned images were acquired using Avatar software.

### Keyes scoring method for evaluating caries

Following micro‑CT acquisition, the samples were rinsed overnight in water to wash away residual PFA. The samples were subsequently stained overnight with 0.4% (w/v) murexide solution (Solarbio, China). The stained molars were then sectioned mesiodistally with a low‑speed precision saw to expose the pulp chambers, allowing magnified observation and recording of the Keyes scores. In accordance with the Keyes criteria [40], lesion severity was graded as follows: E (restricted to enamel), Ds (≤1/4 dentin), Dm (1/4–3/4 dentin), and Dx (>3/4 dentin). Two independent, blinded evaluators performed the scoring; when the discrepancy between the two evaluators exceeded one grade, a third reviewer was consulted, and the final scores were determined by consensus between the two closest evaluations. Data analysis was performed based on each animal’s Keyes score. The data were analyzed using one-way ANOVA with GraphPad Prism 9 (GraphPad Software, USA).

### Isolation of *S. mutans* MVs

MVs were isolated as described in previous studies [41]. Briefly, *S. mutans* 18, UA159, and 593 were cultured in 800 mL of BHI broth for 16 h. Successive centrifugation at 6,000 × *g* and 10,000 × *g* (for 15 min each) was used to remove bacterial cells and debris. Supernatants underwent filtration using a 0.22 μm sterile vacuum filter (Bioland, China). The filtered samples were then subjected to ultracentrifugation (100,000 × *g* for 70 min at 4 °C; Optima XE-100; Beckman Coulter, USA; SW32Ti rotor) to obtain MVs. After being washed twice with sterile PBS, the MVs were resuspended in 500 μL of sterile PBS.

### Characterization of *S. mutans* MVs

The particle size distribution and zeta potential of the MVs were analyzed by nanoparticle tracking analysis (NTA; NanoSight NS300, Malvern Panalytical, UK) and a nanoparticle zeta potential analyzer (ZetaSizer Pro, Malvern Panalytical, UK).

The morphology was examined by transmission electron microscopy (TEM; Hitachi, Japan). Briefly, a 10 μL aliquot of MVs was deposited on copper grids for negative staining with 2% uranyl acetate.

MV yield was normalized based on particle number per CFU (particles/CFU) and total protein content per CFU (pg/CFU). Total protein content was measured using a BCA protein assay kit (Mishu Bio, China).

Data were obtained from three independent biological replicates of MV extraction (*n* = 3).

### Biofilm formation assay

Biofilm formation was quantified using a crystal violet (CV) staining assay according to an earlier report [31]. Briefly, log-phase *S. mutans* UA159 Δ*gtfBC* cultures (OD_600_ = 0.2) were inoculated into 96-well polystyrene plates at a 1:20 volume dilution. Each well was filled with 200 μL of BHI broth supplemented with 1% sucrose and either PBS (negative control, NC) or MVs at different concentrations (0.6, 1.3, and 2.5 μg/mL). After incubation for 24 h, the planktonic cells were removed. The wells were then rinsed three times with PBS. After 30 min of air drying at 37 °C, the biofilms were subjected to 15 min of staining with 0.1% crystal violet solution. Excess dye was removed by washing, and the bound crystal violet was eluted in 95% ethanol. The absorbance was measured at OD_595_ using a microplate reader (SpectraMax iD3; Molecular Devices, USA). The data are from three independent biological replicates.

### Adhesion assay

Bacterial adhesion was evaluated using a glass test tube assay according to the protocol described by Du et al. [14]. Briefly, log-phase *S. mutans* UA159 Δ*gtfBC* cultures (OD_600_ = 0.2) were mixed with MVs (0.6 μg/mL) or PBS (NC) at a 1:20 (v/v) ratio in BHI media supplemented with 1% sucrose (Tube A) and incubated at 37 °C for 24 h under anaerobic conditions with the tubes inclined at 30°.

After incubation, nonadherent bacteria were collected by gently inverting Tube A and transferring the supernatant to a new tube (Tube B). Cells in Tube B were harvested by centrifugation, washed with PBS, and resuspended in PBS. Adherent bacteria remaining in Tube A were detached by adding an equal volume of PBS and vortexing vigorously. The OD_600_ of both adherent and nonadherent fractions was measured using a SpectraMax iD3. The adhesion rate was calculated as follows:

Adhesion rate (%) = OD_600_ (adherent bacteria)/[OD_600_ (adherent bacteria) + OD_600_ (nonadherent bacteria)] × 100%. The data are from three independent biological replicates.

### Colony-forming unit (CFU) counts

A modified colony-forming unit (CFU) count method was used to quantify viable bacteria within the biofilm [38]. The experimental groups were the same as those described above. After the cells were washed with PBS, an equal volume of PBS was added to each well to fully resuspend the biofilm. Cells were subjected to serial 10-fold dilutions ranging from 10^−1^ to 10^−10^, and 100-μL aliquots of each dilution were plated onto BHI agar plates. After anaerobic incubation, the colonies were counted. The data are from three independent biological replicates.

### eDNA assay

Biofilm construction and treatment followed previously described protocols. In brief [38], the biofilms were collected in 1 mL of PBS and centrifuged at 4,000 × *g* for 5 min, after which the supernatants were filtered (0.22 μm) and incubated with SYTOX Green for 30 min to measure the amount of eDNA. The pellets were used for CFU enumeration. To normalize for biomass, eDNA levels after MV treatment were expressed as the ratio of fluorescence intensity to CFU counts. The data are from three independent biological replicates.

### Scanning electron microscopy

Scanning electron microscopy (SEM; ZEISS Sigma 360, Germany) was performed as previously described [38]. Biofilms grown on glass coverslips in 24-well plates for 24 h were washed with PBS, fixed with 2.5% glutaraldehyde overnight at 4 °C, dehydrated through a graded ethanol series, critical-point dried, sputter-coated with gold, and examined by SEM to assess biofilm architecture. The data are from three independent biological replicates.

To visualize MV budding and release on the bacterial surface [25], *S. mutans* 18, UA159, and 593 were cultured for 16 h, and the cell pellets were collected by centrifugation at 4,000 × *g* for 5 min. The pellets were subjected to fixation, dehydration and critical‑point drying, followed by sputter coating with gold before imaging.

### Biofilm analysis and structural imaging

A modified method was used to assess the three-dimensional structure of the biofilms [42]. Briefly, bacterial suspensions were transferred to 15 mm glass-bottom confocal dishes (Biosharp, China), and 1 μM Alexa Fluor 647–conjugated dextran (Invitrogen, USA) was added to label extracellular polysaccharides (EPSs). After biofilm formation occurred under facultative anaerobic conditions, the planktonic cells were removed. Biofilms were then stained with 2.5 μM SYTO 9 (Invitrogen, USA) for 15 min and washed twice.

We obtained three‑dimensional biofilm micrographs on an Olympus confocal laser scanning microscope (CLSM) fitted with a 20 × objective. The detection ranges for the emission signals were adjusted to 495–515 nm (SYTO 9) and 655–690 nm (Alexa Fluor 647). Three independent biological replicates were performed, with three random fields imaged for each sample. Imaris software v9.0.1 (Bitplane, Oxford Instruments, UK) was used to convert serial optical sections into rendered 3D biofilm architectures. The biomass of bacteria and EPS within the biofilms was subsequently quantified.

### Quantification of extracellular polysaccharides

Extracellular polysaccharide extraction and quantification were performed based on a published protocol with minor adjustments [14]. Biofilms were constructed as described above, and the experimental groups were the same as those described above. After being washed with PBS, the biofilm material was dispersed by forceful pipetting and transferred to 1.5 mL microcentrifuge tubes. After centrifugation at 6,000 × *g* for 10 min at 4 °C, the resulting supernatant was the water-soluble polysaccharide (WSP) fraction. This WSP recovery step (PBS resuspension followed by centrifugation) was performed in triplicate to fully elute soluble polysaccharides.

After WSP collection, the remaining residue was redispersed in 1 M NaOH and incubated at 37 °C for 1 h to extract the water-insoluble polysaccharide (WIP). Each polysaccharide extract was blended with three volumes of anthrone‑sulfuric acid reagent. The samples were incubated at 95 °C for 6 min before they were cooled to room temperature. Next, 200 μL of each reaction mixture was transferred into 96‑well plates for detection. The absorbance was measured at 625 nm, and polysaccharide concentrations were calculated using a glucose standard curve. The data are from three independent biological replicates.

### Acid production assay

Acid production capacity was assessed as an indicator of biofilm metabolic activity. Following a previously described method with modifications [14], the acidogenicity of MV-induced *S. mutans* UA159 Δ*gtfBC* biofilms was evaluated. Briefly, the supernatants from 24 h biofilm cultures were collected and centrifuged at 4,000 × *g* for 5 min at room temperature to remove cellular debris. The pH of the clarified supernatants was recorded with a digital pH meter (Thermo Fisher, USA). The data are from three independent biological replicates.

### Quantitative real-time PCR analysis

Quantitative real-time polymerase chain reaction (RT‒qPCR) was performed to evaluate the expression of biofilm-related genes, with modifications to a previously described protocol [14]. The experimental groups were identical to those described above. Bacterial suspensions were cultured in 24-well plates, and biofilms were harvested after 24 h, stabilized with RNAprotect Bacteria Reagent (Qiagen, USA), and stored at −80 °C until analysis.

Total bacterial RNA was isolated using an RNA extraction kit manufactured by Qiagen (USA). After the removal of genomic DNA, reverse transcription for cDNA synthesis was performed with the PrimeScript™ RT Reagent Kit with gDNA Eraser (Takara Bio, Japan). RT‒qPCRs were conducted on a LightCycler® 480 Real‑Time PCR System (Roche, Switzerland) with SYBR® Premix Ex Taq™ (Tli RNaseH Plus) (Takara Bio, Japan). The expression levels of *ldh, gtfB, and gtfC* were normalized to those of 16S rRNA. The primer sequences are available in Table S2. Relative gene expression was quantified with the 2^−ΔΔCt^ method. The data are from three independent biological replicates.

### Astral-DIA proteomic analysis

To eliminate contaminating proteins, the crude MV pellet was purified by iodixanol density gradient centrifugation [43]. Briefly, the sample was loaded onto the top of a step gradient (5%, 10%, 20%, and 40% iodixanol; OptiPrep, Sigma, USA) and centrifuged at 200,000 × *g* for 3 h at 4 °C. The fraction at the 20%–40% interface was collected, washed with PBS, and re‑centrifuged. The purified MVs were resuspended in PBS and stored at −80 °C until use.

A total of 6 samples were analyzed (3 biological replicates per strain for ^*S. mutans* 18^MVs and ^*S. mutans* 593^MVs) [44]. Proteins were extracted from MVs using SDT (4% SDS, 100 mM Tris-HCl, pH 7.6) lysis buffer, and protein concentrations were determined with a BCA protein assay kit (Beyotime, China). Each sample containing 15 μg of protein was analyzed by SDS‑PAGE (4–20% gradient gel, 180 V, 45 min) for quality verification, followed by filter‑aided sample preparation (FASP) for tryptic digestion. After digestion, the peptides were desalted on Empore SPE Cartridges MCX (30 μm, Waters). Desalted peptides were lyophilized by vacuum centrifugation and reconstituted in 0.1% formic acid, and their concentrations were determined using a peptide assay. iRT standard peptides were added to each peptide sample and analyzed by nanoliquid chromatography (Vanquish Neo, Thermo Scientific) coupled online to an Orbitrap Astral mass spectrometer (Thermo Scientific) operated in data‑independent acquisition (DIA) mode. MS1 scans covered m/z 380–980 at a resolution of 240,000; DIA‑MS2 used 299 isolation windows with a collision energy of 25 eV. DIA data were processed using DIA-NN 1.8.1 against the UniProt database, with a false discovery rate (FDR) < 1% for both peptide and protein identification. Only proteins detected in at least half of the replicates per group were included in the quantitative analysis.

For principal component analysis, R Base (v 4.3.1) was used to perform principal component analysis (PCA), and linear dimension reduction was applied to tens of thousands of protein quantification variables to extract the principal components PC1, PC2, and PC3, along with their corresponding contribution rates.

Differentially expressed proteins (DEPs) were identified using limma (v 3.56.2). Proteins exhibiting (| log_2_(fold change) | > 1) together with *P-*adjust < 0.05 were classified as significant DEPs, including upregulated and downregulated proteins. Volcano plots were generated using the ggplot2 package, with log_2_ (fold change) on the x‑axis and −log_10_ (*P* value) on the y‑axis; red points indicate significantly upregulated proteins, blue points indicate significantly downregulated proteins, and gray points indicate proteins with no significant difference. Data normalization and differential expression analysis were conducted on the Wekemo Bioincloud platform (https://www.bioincloud.tech/).

### Bioinformatics analysis

The functional annotation and enrichment of the identified proteins were performed according to previously published procedures [45]. Gene Ontology (GO) annotations were obtained by running EggNOG‑mapper (v2.0.1) against the EggNOG 5.0 reference database. The proteins were subsequently categorized based on the three main GO domains: cellular component (CC), molecular function (MF), and biological process (BP). Pathway annotations were obtained by aligning protein sequences against the Kyoto Encyclopedia of Genes and Genomes (KEGG) pathway database to assign pathway IDs. Hypergeometric tests were conducted using clusterProfiler (v4.8.1), with the entire set of KEGG-annotated proteins as the background, to identify pathways significantly enriched among the DEPs. GO terms and KEGG pathways with *P-*adjust *<* 0.05 were considered to be significantly enriched.

The subcellular localization of the identified proteins was predicted using the PSORTb, CELLO, AureoWiki and UniProt databases [46].

### Coomassie blue staining

Total protein was extracted from 16 h planktonic cultures using a Bacterial Protein Extraction Kit (Solarbio, China) according to the manufacturer’s instructions [47]. Protein extracts and MV preparations were separately mixed with an equal volume of 5 × protein loading dye (NCM Biotech, China) and heated at 95 °C for 5 min. Equal amounts of protein (10 μg/well) were then resolved by SDS‒PAGE on a 12% polyacrylamide gel. The protein bands were visualized by staining with Coomassie Brilliant Blue (Epizyme Biotech, China). The data are from three independent biological replicates.

### Inhibition of enzymatic activity and cell-free catalytic assays

To determine whether MV-mediated EPS synthesis depends on vesicle-associated enzymatic activity, experiments were performed with modifications to a previously described protocol [48,49]. ^*S. mutans* 593^MVs were heat-inactivated at 70 °C for 0, 30, 60, or 90 min. Heat-inactivated (70 °C for 30, 60, or 90 min) or untreated (0 min) ^*S. mutans* 593^MVs (0.6 μg/mL) were incubated together with ^*S. mutans* 18^MVs (0.6 μg/mL) and the UA159 Δ*gtfBC* in BHIS medium for 24 h. PBS was used in place of MVs as the negative control. Biofilm biomass was quantified by CV staining, and WSP and WIP were quantified using the anthrone-sulfuric acid assay.

To assess the intrinsic catalytic activity of MVs, a cell-free assay was performed. Heat-inactivated (70 °C, 30 min) or untreated ^*S. mutans* 593^MVs (10 μg/mL) were incubated together with ^*S. mutans* 18^MVs (10 μg/mL) in BHIS medium at 37 °C for 24 h in the absence of bacterial cells. PBS served as the negative control. The formation of flocculent aggregates was monitored, and WSP and WIP were quantified using the anthrone-sulfuric acid assay. The data are from three independent biological replicates.

The proteinase K treatment of MVs was performed according to a previously described method with minor modifications [50]. Briefly, MVs were incubated with Proteinase K at a final concentration of 100 μg/mL (Tiangen Biotech Co., Ltd., China) for 1 h at room temperature. The digestion reaction was terminated by the addition of 50 × protease inhibitor cocktail (Beyotime, China). The mixture was ultracentrifuged at 100,000 × g for 70 min at 4 °C, after which the pellet was washed twice with PBS. The data are from three independent biological replicates.

### Statistical analysis

The results are expressed as the mean ± standard deviation (SD) from at least three biological replicates and three technical replicates. GraphPad Prism 9 (GraphPad Software, USA) was used for all the statistical analyses. Two‑way repeated‑measures ANOVA was used to evaluate the growth curve data. Multigroup comparisons were performed using one-way ANOVA followed by Dunnett’s or Tukey’s post hoc tests. *P* < 0.05 was considered statistically significant.

## Results

To investigate the relationship between MV heterogeneity and strain-specific cariogenicity, we first established the cariogenic phenotypes of the parental strains in vivo, followed by comparative characterization of MV physicochemical properties, functional assays in a recipient bacterial background, proteomic profiling of MV cargo, and, finally, cell-free experiments to assess MV activity independent of viable bacterial cells.

### *S. mutans* 593 exhibits the strongest cariogenic capacity

To compare the strain-dependent cariogenic potential of *S. mutans*, two clinical oral isolates, designated *S. mutans* 18 and *S. mutans* 593, were obtained from patients with distinct caries experience and were selected together with the reference strain UA159. The species identity of the two clinical isolates was first evaluated by 16S rRNA gene sequence analysis. Phylogenetic analysis revealed that both isolates grouped closely with UA159 in the phylogenetic tree and that the 16S rRNA gene sequence was 100% identical to that of UA159, which confirmed their taxonomic assignment to *S. mutans* (Fig. S1A). Gram staining further confirmed that all three strains were Gram-positive cocci arranged in typical chains, with chain length increasing in the order *S. mutans* 18, UA159, and *S. mutans* 593 (Fig. S1B). Compared with the reference strain UA159, both clinical isolates exhibited more vigorous growth and entered the logarithmic growth phase earlier (Fig. S1C; *P* < 0.05).

After the identity and basic biological characteristics of the strains were established, a rat dental caries model was established to evaluate the in vivo cariogenicity of these strains (Figure 1A). Throughout the experimental period, body weight gain was comparable among all groups, with no statistically significant differences observed (Figure 1B), indicating that bacterial inoculation did not markedly affect the general growth status of the animals. Compared with those in the NC group, the bacterial colonization levels in the rat molar region increased progressively in the order of *S. mutans* 18, UA159, and *S. mutans* 593 (Figure 1C; *P* < 0.05).

**Figure 1.**
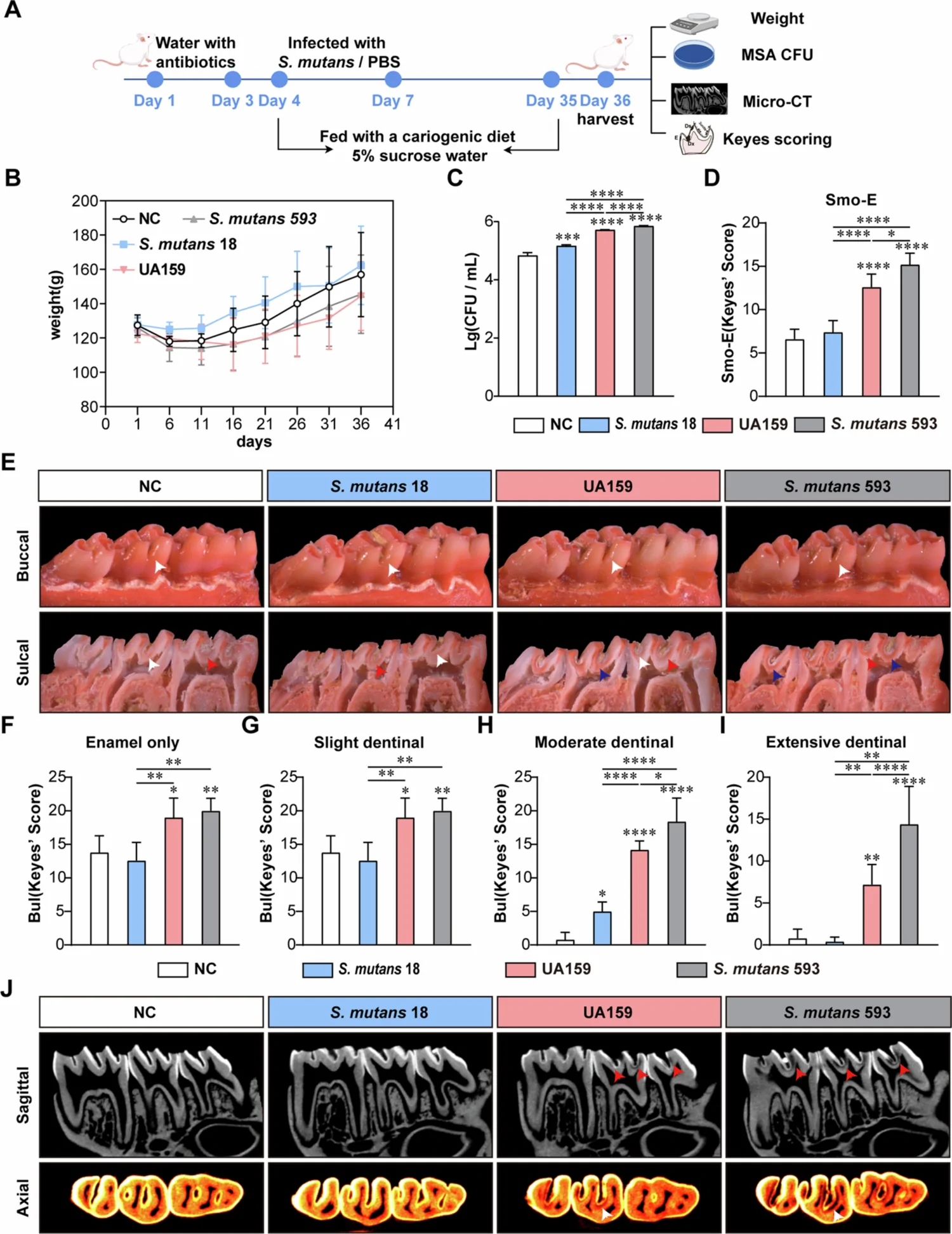
Comparative cariogenicity of *S. mutans* strains. (A) Schematic of the experimental procedures for establishing the dental caries model. (B) Body weight changes recorded throughout the experimental period. (C) *S. mutans* CFU counts on mandibular tooth surfaces after sacrifice. (D) Keyes scores for smooth‑surface lesions. (E) Representative photographs of murexide‑stained carious lesions on buccal and sulcal surfaces (buccal views: white arrows indicate lesions; sulcal views: white, red, and blue arrows indicate slight, moderate, and extensive dentinal involvement, respectively). (F–I) Sulcal surface scores categorized by lesion depth and extent. (J) Micro‑CT scans of molars (sagittal views: red arrows indicate carious defects; coronal views: white arrows indicate carious lesions). NC, negative control group (PBS‑treated). The data are presented as the mean ± SD (*n* = 5 rats per group). *P < 0.05, **P < 0.01, ***P < 0.001, ****P < 0.0001.

Caries scoring revealed that compared with the NC group, all three *S. mutans*-inoculated groups had significantly higher enamel lesion scores on smooth surfaces, with the *S. mutans* 593 group exhibiting the highest enamel lesion scores (Figure 1D, E; *P* < 0.05). Given that pit-and-fissure sites are highly susceptible to early carious lesions, pit-and-fissure caries were further assessed according to lesion incidence and severity. Lesions were categorized as total lesions (E + Ds + Dm + Dx), restricted to enamel (E), slight dentinal lesions (Ds), moderate dentinal lesions (Dm), and extensive dentinal lesions (Dx). Total lesion scores were used to reflect caries incidence, whereas the individual lesion categories represented caries severity. The *S. mutans* 593 group showed a significantly higher caries incidence than both the NC and *S. mutans* 18 groups. Moreover, *S. mutans* 593 induced more severe dentinal destruction, as evidenced by higher Dm and Dx scores than those observed in the UA159 group (Figure 1E–I; *P* < 0.05). Micro-CT analysis revealed discontinuous enamel surfaces and low-density radiolucent zones in molars from the UA159 and *S. mutans* 593 groups, whereas these zones were absent or minimal in the *S. mutans* 18 and NC groups (Figure 1J). These findings indicate a strain-dependent increase in cariogenic potential, following the order *S. mutans* 18 < UA159 < *S. mutans* 593.

### *S. mutans* 593 produces high-yield MVs with distinct properties

To investigate the potential association between the cariogenic capacity of these strains and the biological characteristics of their secreted MVs, we isolated MVs derived from three strains: *S. mutans* 593, UA159, and *S. mutans* 18 (designated as ^*S. mutans* 593^MVs, ^UA159^MVs, and ^*S. mutans* 18^MVs, respectively). SEM provided direct visualization of MV biogenesis, revealing that spherical vesicles budded from the bacterial surface (Figure 2A). Marked differences in vesicle morphology and size were observed among the strains. MVs produced by the highly cariogenic strain *S. mutans* 593 were significantly larger, following the order ^*S. mutans* 593^MVs > ^UA159^MVs > ^*S. mutans* 18^MVs (Figure 2A). To further characterize the ultrastructures of the isolated vesicles, TEM revealed that the vesicles exhibited a characteristic bilayered, cup-shaped morphology (Figure 2B).

**Figure 2.**
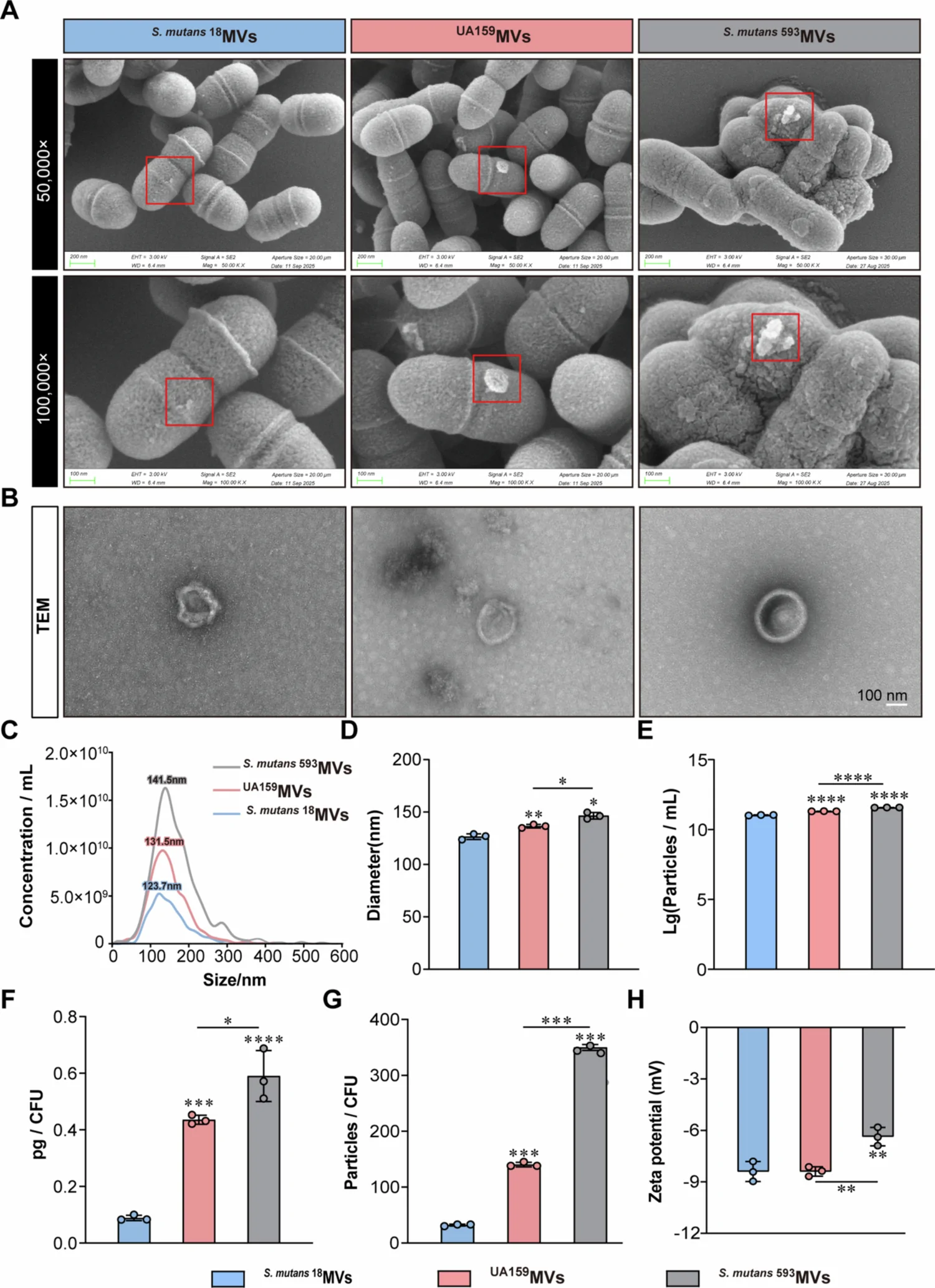
Characterization of MVs derived from *S. mutans* strains with different cariogenic potentials. (A) Representative SEM images showing vesicle budding from the bacterial cell surface at 50,000 × and 100,000 × magnification (scale bars: 200 nm and 100 nm, respectively). (B) Representative TEM images of MVs (scale bar: 100 nm). (C) The size distribution of MVs measured by NTA. (D) The mean particle diameter was analyzed by NTA. (E) Particle concentration of MVs determined by NTA. (F) MV-associated protein yield was normalized to the number of CFUs and quantified by BCA assay. (G) MV particle yield normalized to the number of CFUs. (H) Zeta potential of MVs. Data are presented as the mean ± SD from three independent biological replicates (*n* = 3). *P < 0.05, **P < 0.01, ***P < 0.001, ****P < 0.0001.

NTA revealed distinct differences in the size distribution and particle concentration of MVs derived from the three strains (Figure 2C). The mean particle diameters of the ^*S. mutans* 593^MVs, ^UA159^MVs, and ^*S. mutans* 18^MVs were 144.9 ± 4.3 nm, 131.6 ± 6.7 nm, and 126.6 ± 2.7 nm, respectively (Figure 2D; *P* < 0.05). In parallel, their mean particle concentrations were 3.77 × 10^11^ ± 4.71 × 10^9^, 2.03 × 10^11^ ± 4.71 × 10^9^, and 1.07 × 10^11^ ± 5.77 × 10^9^ particles/mL, respectively (Figure 2E; *P* < 0.05). Both the MV diameter and the particle concentration increased in parallel with the cariogenic potency of the corresponding strains, suggesting that highly cariogenic *S. mutans* strains may possess enhanced vesicle production capacity.

To exclude the influence of differences in initial bacterial biomass during MV isolation, the MV yield was normalized to the CFU. The results of the BCA assay revealed that the amount of MV-associated protein per CFU in the ^*S. mutans* 593^MV group was 1.8-fold greater than that in the ^UA159^MV group and 2.2-fold greater than that in the ^*S. mutans* 18^MV group. In addition, the ^UA159^MV group exhibited a 1.2-fold greater MV-associated protein yield per CFU than did the ^*S. mutans* 18^MV group (Figure 2F; *P* < 0.05). Consistent with these findings, the ^*S. mutans* 593^MV group released the greatest number of MV particles per CFU (Figure 2G; *P* < 0.05). Zeta potential analysis revealed that all the MVs were negatively charged. MVs derived from *S. mutans* 593 exhibited a less negative zeta potential than did those derived from UA159 and *S. mutans* 18, indicating a greater propensity to aggregate (Figure 2H; *P* < 0.05).

These physicochemical characterization results indicate that the highly cariogenic strain *S. mutans* 593 exhibits a pronounced high-MV-yield phenotype. Differences in particle size, vesicle yield, and surface properties may provide a structural basis for its functional advantages within the dental plaque microenvironment.

### MVs restore cariogenic phenotypes in a cariogenicity-dependent manner

Gram staining revealed that compared with the wild-type UA159 strain, UA159 Δ*gtfBC* retained the typical Gram-positive coccoid morphology but formed shorter chains and exhibited a looser cellular arrangement (Fig. S2A). RT‒qPCR confirmed the complete loss of *gtfBC* transcription in the mutant (Fig. S2B; *P* < 0.05). Although the mutant entered the exponential phase slightly later than the wild type did, its overall growth was comparable (Fig. S2C). These results confirmed the loss of *gtfB*/*C* expression in UA159 Δ*gtfBC*, which is consistent with the functional phenotype previously verified for this strain ^[51]^, without markedly affecting bacterial viability.

We next examined whether MVs from *S. mutans* strains with different cariogenic potentials could rescue the impaired phenotype of the mutant. CV staining revealed that exogenous MVs restored biofilm formation in a concentration-dependent manner. Among the concentrations tested, 0.6 μg/mL resulted in the greatest difference among the MV groups. At this concentration, the biofilm biomass increased in the order of ^*S. mutans* 18^MVs < ^UA159^MVs < ^*S. mutans* 593^MVs (Figure 3A; *P* < 0.05). This dose-dependent trend is consistent with previously reported crystal violet results for UA159 MVs applied to the UA159 Δ*gtfBC* biofilm [28]. On the basis of these data, 0.6 μg/mL was selected as the working concentration for the subsequent in vitro functional experiments. To verify whether the functional differences resulted from intrinsic differences in MV activity, biofilm formation was assessed at the same concentration (2 × 10^7^ particles/mL), and the trend revealed by CV staining was consistent with that previously observed when MVs were applied at a protein concentration of 0.6 μg/mL (Fig. S3A, B; *P* < 0.05).

**Figure 3.**
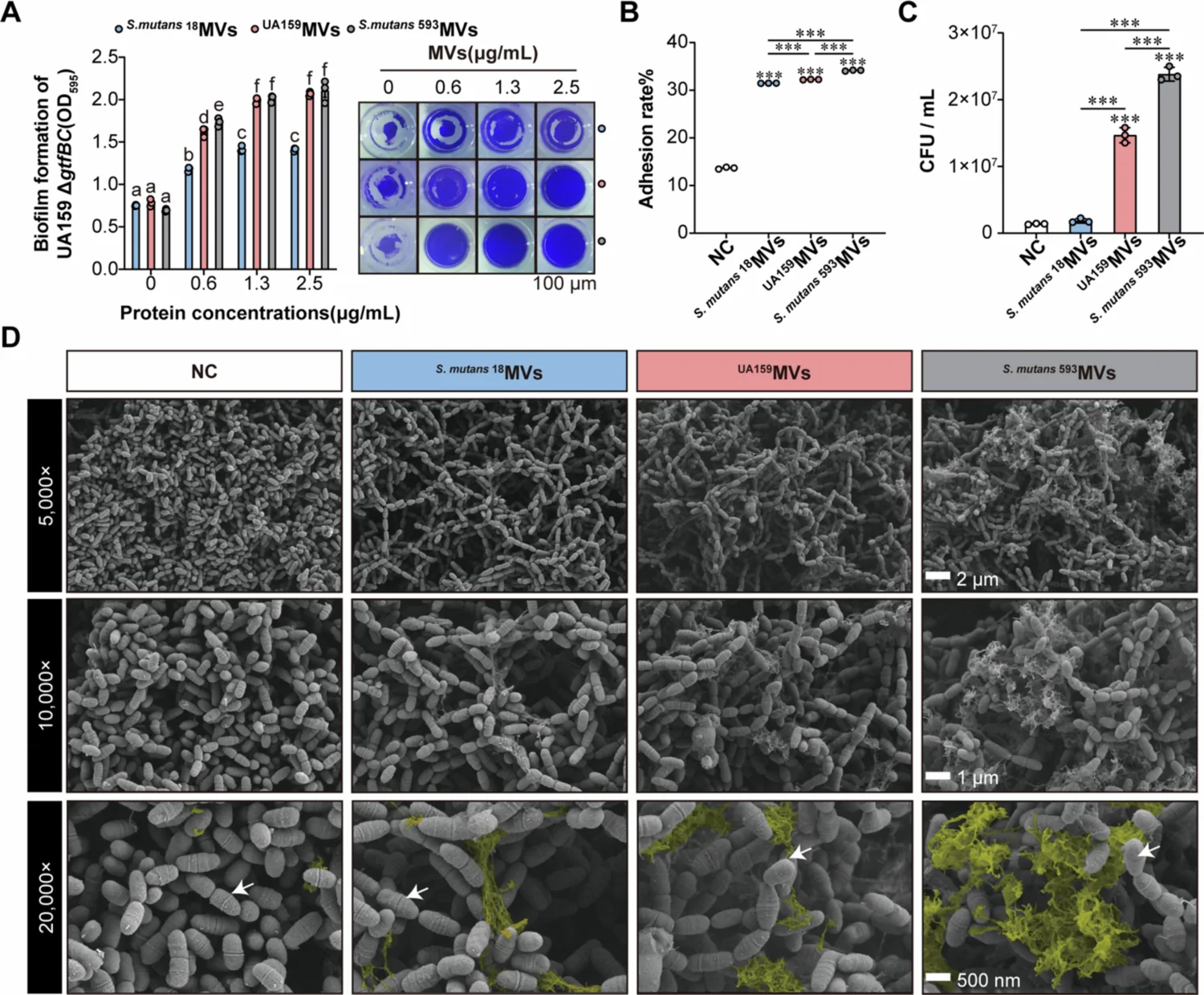
Comparison of MV functions across strains with different cariogenic potentials. (A) The concentration-dependent restoration of biofilm formation by exogenous MVs was assessed by crystal violet (CV) staining. Different letters indicate significant differences (*P* < 0.05). (B) Initial adhesion of UA159 Δ*gtfBC* to glass surfaces following MV treatment. (C) Viable bacterial counts within biofilms determined by a colony-forming unit (CFU) count assay. (D) Representative SEM images of 24-h biofilms at 5,000×, 10,000×, and 20,000× magnification (scale bars: 2 μm, 1 μm, and 500 nm, respectively). White arrows indicate bacterial cells, and the pseudocolored yellow regions represent the extracellular matrix. NC, negative control (UA159 Δ*gtfBC* treated with PBS); ^*S. mutans* 18^MVs, UA159 Δ*gtfBC* treated with *S. mutans* 18-derived MVs; ^UA159^MVs, UA159 Δ*gtfBC* treated with UA159-derived MVs; ^*S. mutans* 593^MVs, UA159 Δ*gtfBC* treated with *S. mutans* 593-derived MVs. The data are presented as the mean ± SD from three independent biological replicates (*n* = 3). *P < 0.05, **P < 0.01, ***P < 0.001, ****P < 0.0001.

MVs also restored multiple biofilm-related phenotypes in a strain-dependent manner. Adhesion analysis revealed that MV treatment significantly increased the initial adhesion of the mutant to glass surfaces, with ^*S. mutans* 593^MVs having the strongest effect (Figure 3B; *P* < 0.05). Similarly, MVs significantly increased the viable bacterial population within the biofilms, following the same trend (^*S. mutans* 18^MVs < ^UA159^MVs < ^*S. mutans* 593^MVs) (Figure 3C).

To determine whether MVs altered biofilm architecture, 24-h biofilms were analyzed by SEM and CLSM. Compared with the NC group, all the MV-treated groups developed a three-dimensional extracellular matrix with an interconnected network structure, whereas the bacterial morphology remained unchanged (Figure 3D). Among the three MV groups, ^*S. mutans* 593^MVs produced the most compact extracellular matrix, which tightly surrounded bacterial cells. CLSM analysis further supported the observation that MV treatment increased bacterial biomass within the biofilm (Figure 4A, B; *P* < 0.05) and markedly promoted EPS production (Figure 4A, C; *P* < 0.05).

**Figure 4.**
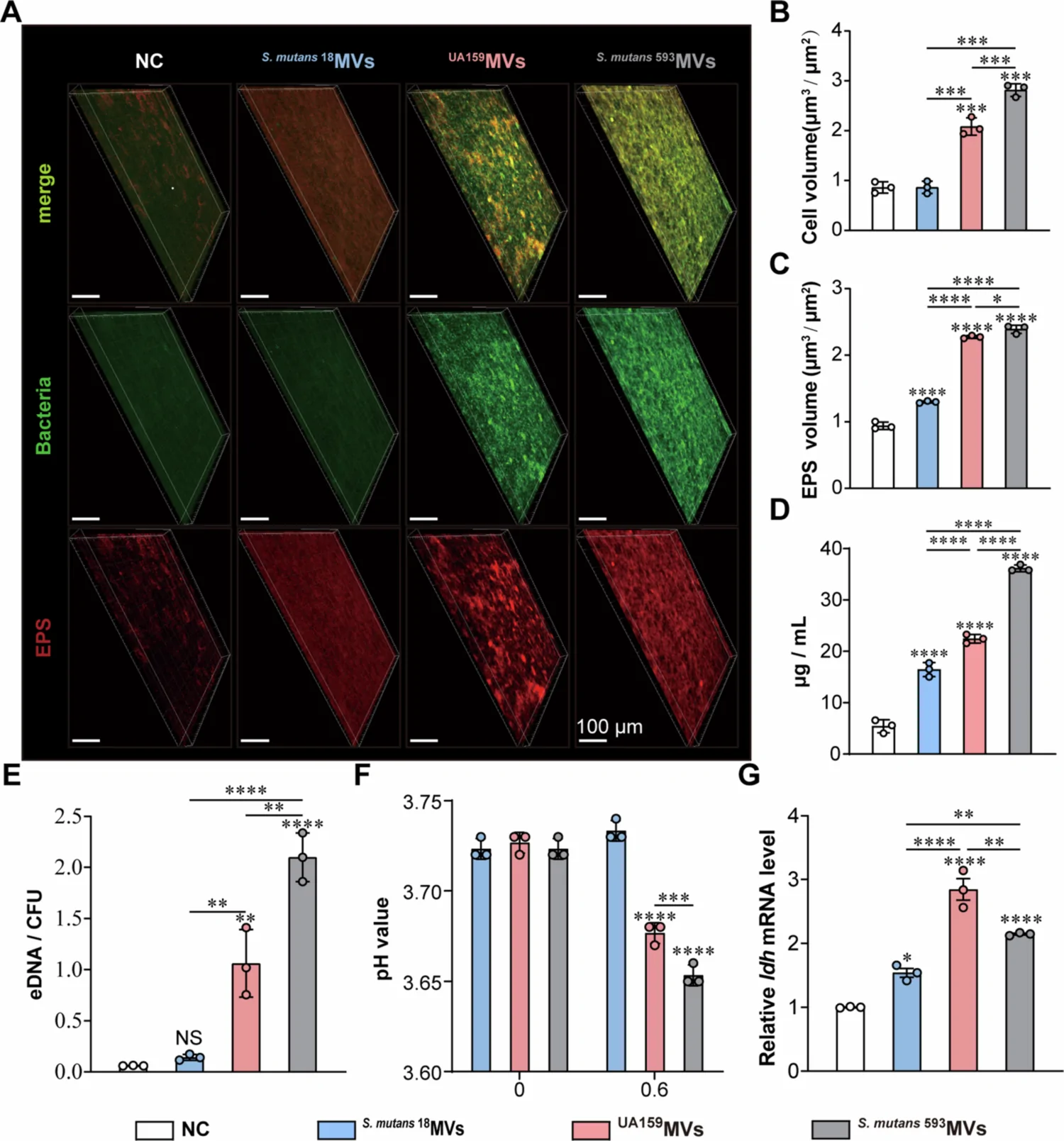
Comparison of MV functions across strains with different cariogenic potentials. (A) Representative CLSM images and Imaris-generated three-dimensional reconstructions of 24-h biofilms. Bacterial cells were stained with SYTO 9 (green), and extracellular polysaccharides (EPS) were stained with Alexa Fluor 647 (red). Scale bar: 100 μm. (B) Quantification of bacterial biovolume. (C) Quantification of EPS biovolume. Both analyses were performed using Imaris software. (D) Quantification of water-insoluble polysaccharide (WIP) using the anthrone–sulfuric acid assay. (E) Quantification of eDNA. (F) Biofilm acidogenicity was assessed by an acid production assay. (G) RT‒qPCR detection of the expression of the acid-producing gene *ldh*. NC, negative control (UA159 Δ*gtfBC* treated with PBS); ^*S. mutans* 18^MVs, UA159 Δ*gtfBC* treated with *S. mutans* 18-derived MVs; ^UA159^MVs, UA159 Δ*gtfBC* treated with UA159-derived MVs; ^*S. mutans* 593^MVs, UA159 Δ*gtfBC* treated with *S. mutans* 593-derived MVs. Data are presented as the mean ± SD from three independent biological replicates (*n* = 3). *P < 0.05, **P < 0.01, ***P < 0.001, ****P < 0.0001.

Quantitative analysis revealed distinct differences in EPS composition among the MV groups (Figure 4D; *P* < 0.05). WIP, the major structural component responsible for biofilm stability, increased progressively from ^*S. mutans* 18^MVs to ^UA159^MVs and reached the highest level in the ^*S. mutans* 593^MV group, a trend consistent with that observed for particle concentration (Fig. S3C; *P* < 0.05). In the MV-treated groups, eDNA accumulation in the biofilm increased in parallel with the cariogenic potential of the source strains (Figure 4E; *P* < 0.05).

We further investigated whether these structural alterations were accompanied by changes in metabolic activity. Compared with the NC group, ^*S. mutans* 18^MVs did not significantly affect acid production. In contrast, ^UA159^MVs and ^*S. mutans* 593^MVs significantly enhanced biofilm acidogenicity, with ^*S. mutans* 593^MVs exerting the most pronounced effect (Figure 4F; *P* < 0.05), a trend consistent with that observed for particle concentration (Fig. S3D; *P* < 0.05). These findings were consistent with the RT‒qPCR results, indicating that MVs derived from highly cariogenic strains, but not those from low-cariogenic strains, may increase biofilm acidogenicity by modulating the transcription of *ldh* (Figure 4G; *P* < 0.05).

These findings indicate that exogenous MVs can restore impaired biofilm formation and metabolic activity in UA159 Δ*gtfBC* in a manner closely associated with the cariogenicity of the source strain, suggesting that MVs from highly cariogenic strains may have greater potential to promote dental caries.

### Highly cariogenic *S. mutans* strains produce MVs enriched with virulence factors

To examine the MV composition and cargo in more detail, the purified MVs were analyzed by Orbitrap Astral data-independent acquisition (DIA)-based quantitative proteomics. PCA demonstrated excellent reproducibility among biological replicates and clear separation of the MV groups (Figure 5A), indicating distinct proteomic profiles across strains. A total of 1,601 proteins were identified, including 1,290 core proteins shared by all the strains (Figure 5B). ^*S. mutans* 18^MVs contain 62 unique proteins, whereas ^*S. mutans* 593^MVs contain 249, which is significantly more than the number in ^*S. mutans* 18^MVs.

**Figure 5.**
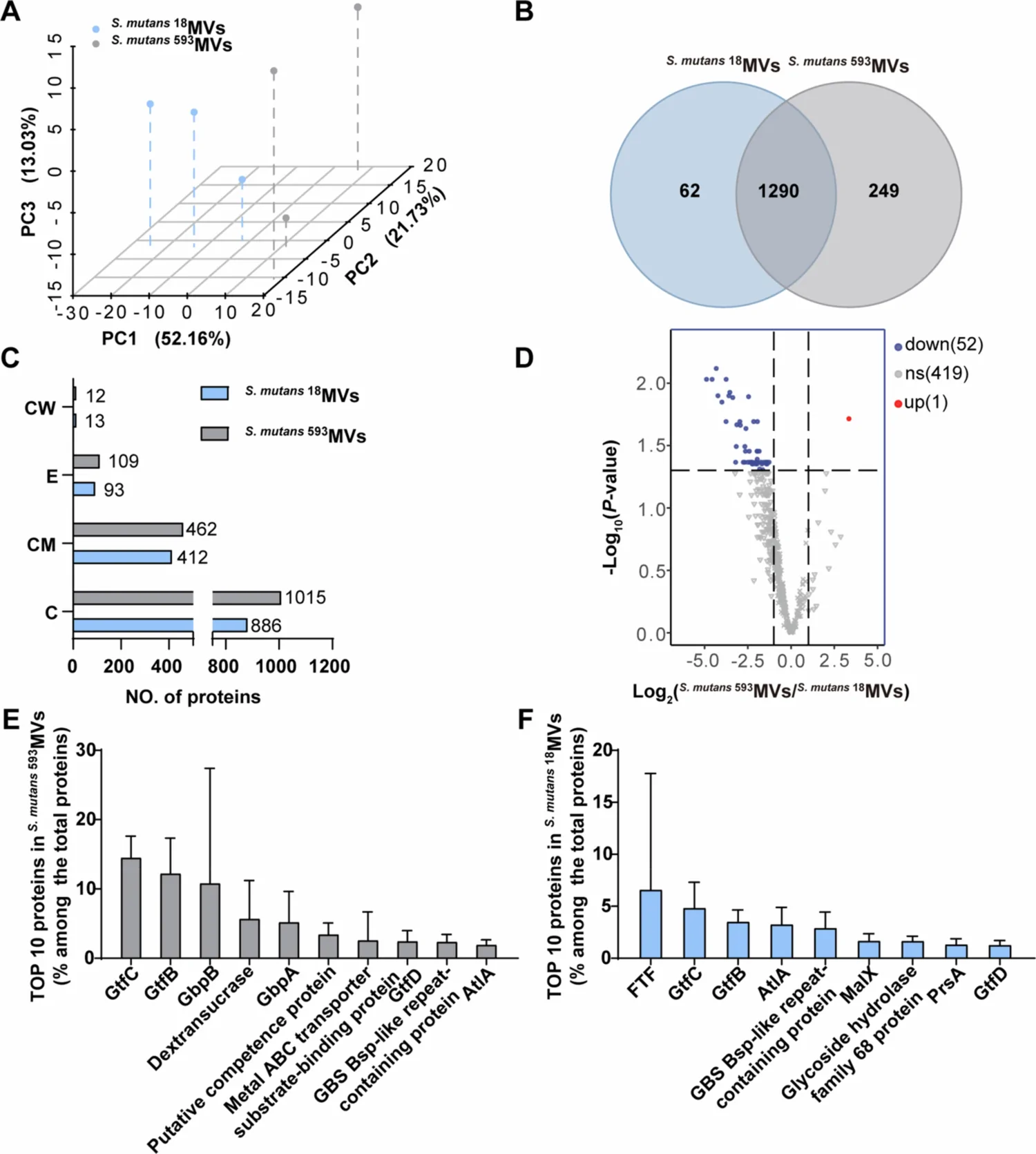
Quantitative proteomics characterization of MVs from *S. mutans* strains with different cariogenic potentials. (A) Principal component analysis of MV proteomic profiles, showing high reproducibility among biological replicates and clear separation between the two experimental groups. (B) Venn diagram showing the shared and unique proteins identified in ^*S. mutans* 18^MVs and ^*S. mutans* 593^MVs. (C) Predicted subcellular localization of proteins identified in MVs. CW, cell wall; E, extracellular; CM, cytoplasmic membrane; C, cytoplasm. (D) Volcano plot showing DEPs in ^*S. mutans* 593^MVs versus ^*S. mutans* 18^MVs. Red dots indicate significantly upregulated proteins, blue dots indicate significantly downregulated proteins, and gray dots indicate proteins whose expression did not significantly differ. (E, F) Relative abundance of the top 10 proteins identified in ^*S. mutans* 593^MVs (E) and ^*S. mutans* 18^MVs (F), as determined by DIA-NN software. The data are shown as the mean ± SD of three biological replicates.

Subcellular localization prediction of the proteins loaded into ^*S. mutans* 593^MVs revealed that the majority were cytoplasmic (1015, 63.5%), followed by cell membrane-associated (462, 28.9%), extracellular (109, 6.8%), and cell wall-associated proteins (12, 0.8%) (Figure 5C). The subcellular localization profile of proteins associated with ^*S. mutans* 593^MVs was identical to that of ^*S. mutans* 18^MVs, with proteins predominantly originating from the cytoplasm and cytoplasmic membrane, whereas relatively few were assigned to the extracellular space or cell wall (Figure 5C).

By comparing protein levels between ^*S. mutans* 593^MVs and ^*S. mutans* 18^MVs, 53 differentially expressed proteins (DEPs) were identified. Of these, 1 DEP was significantly upregulated and 52 were downregulated in ^*S. mutans* 593^MVs (Figure 5D; Tables S3 and S4). Moreover, the abundance and relative proportions of the top 10 proteins differed markedly between ^*S. mutans* 593^MVs and ^*S. mutans* 18^MVs. ^*S. mutans* 593^MVs contained higher levels of GTFs (GtfB, GtfC, and GtfD), glucan-binding proteins (GbpA and GbpB), and dextransucrase than did ^*S. mutans* 18^MVs, indicating the preferential enrichment of proteins involved in EPS synthesis and biofilm matrix formation (Figure 5E, F).

We next aimed to characterize the functional landscape of proteins that were differentially incorporated into MVs by performing GO enrichment analysis, thereby revealing distinct functional properties of the differential proteomes. GO annotation classified the identified proteins into three functional categories: BP, CC, and MF (Figure 6A). Although the GO enrichment results were not statistically significant (adjusted *P* > 0.05), exploratory analysis showed that proteins were mainly enriched in growth-related processes (BP), membrane-associated components (CC), and binding activities (MF).

**Figure 6.**
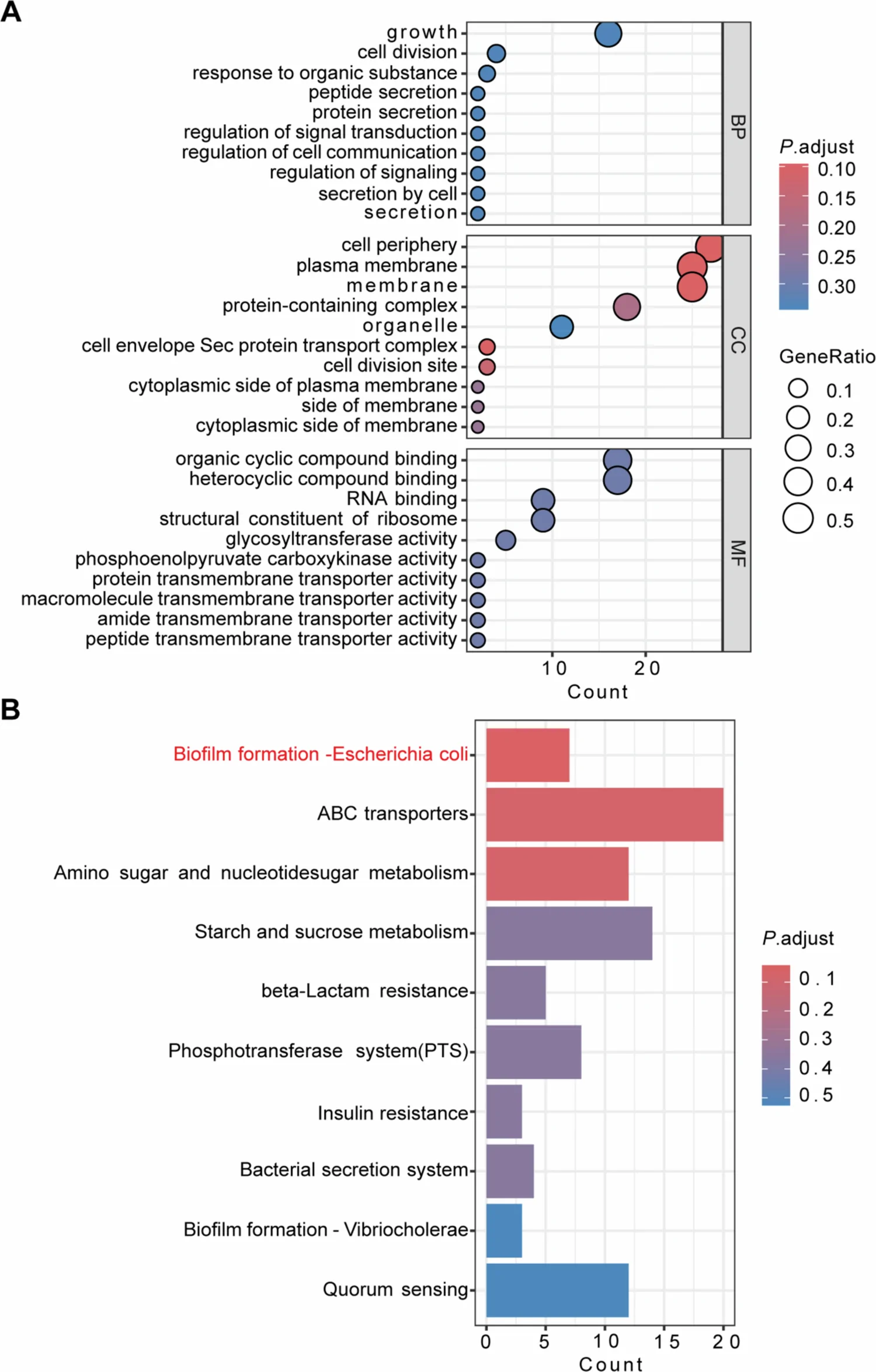
Functional enrichment of MVs from *S. mutans* strains with different levels of cariogenicity. (A) GO enrichment analysis of differentially expressed proteins identified in ^*S. mutans* 593^MVs compared with ^*S. mutans* 18^MVs. Enriched terms are grouped into biological process (BP), cellular component (CC), and molecular function (MF) categories. (B) KEGG pathway enrichment analysis of DEPs in ^*S. mutans* 593^MVs relative to those in ^*S. mutans* 18^MVs. Bubble size indicates the number of enriched proteins, and color indicates the *P*-adjust.

To further explore the functional characteristics, we performed KEGG pathway analysis. KEGG pathway analysis showed that these DEPs were predominantly enriched in “Biofilm formation—Escherichia coli”. In addition, ABC transporters and starch and sucrose metabolism were enriched as exploratory results (adjusted *P* > 0.05) (Figure 6B). Functional annotation indicated these pathways underpin core bacterial activities: intercellular communication, carbohydrate consumption, and nutrient acquisition. These findings suggest that in highly cariogenic strains, proteins involved in protein synthesis, stress response, carbohydrate metabolism, and biofilm formation may share functional links.

### The functional differences among MVs are closely associated with their protein composition

To validate the proteomic findings, SDS–PAGE was performed to compare the protein profiles of cells and MVs derived from the two bacterial strains. Coomassie Brilliant Blue staining revealed no apparent differences in the overall protein profiles of the *S. mutans* 18 and *S. mutans* 593 whole-cell lysates (Fig. S4). In contrast, the MVs released by the two strains displayed markedly distinct protein banding patterns. Compared with ^*S. mutans* 18^MVs, ^*S. mutans* 593^MVs exhibited a greater number of protein bands and pronounced differences in band intensity, which is consistent with the quantitative proteomic results. SDS‒PAGE does not independently validate the differential abundance of the specific proteins identified by quantitative proteomics.

Moreover, proteomic analysis revealed that GTF family proteins were enriched in MVs released by highly cariogenic *S. mutans* 593. Given the heat-labile nature of these proteins [52], we next investigated whether the functional differences between MVs derived from the two strains were primarily attributable to their protein components.

CV staining revealed that heat treatment progressively impaired the ability of ^*S. mutans* 593^MVs to restore biofilm formation in UA159 Δ*gtfBC* (Figure 7A; *P* < 0.05). Compared with that of the untreated MVs (0 min), the biofilm biomass decreased in a heat treatment duration-dependent manner. After 30 min of heating, the biofilm-promoting effect of ^*S. mutans* 593^MVs was reduced to a level comparable to that of ^*S. mutans* 18^MVs. Extending the heat treatment to 90 min nearly abolished their activity, resulting in a biofilm biomass comparable to that observed in the NC group. Consistent with these findings, anthrone–sulfuric acid assays demonstrated that the amounts of both WSP and WIP progressively decreased with increasing heat treatment duration (Figure 7B, C; *P* < 0.05). Following coincubation with proteinase K, MV-mediated biofilm formation (Fig. S3A, B; *P* < 0.05) and WIP accumulation (Fig. S3C; *P* < 0.05) were both suppressed to varying degrees, accompanied by a corresponding increase in the pH of the culture system (Fig. S3D; *P* < 0.05). When UA159 Δ*gtfBC* was supplemented with MVs secreted by UA159 Δ*gtfBC*, no significant change in biofilm biomass was observed (Fig. S4B; *P* < 0.05), suggesting that the protein components of MVs play an important role in the biofilm-promoting and acidogenic phenotypes.

**Figure 7.**
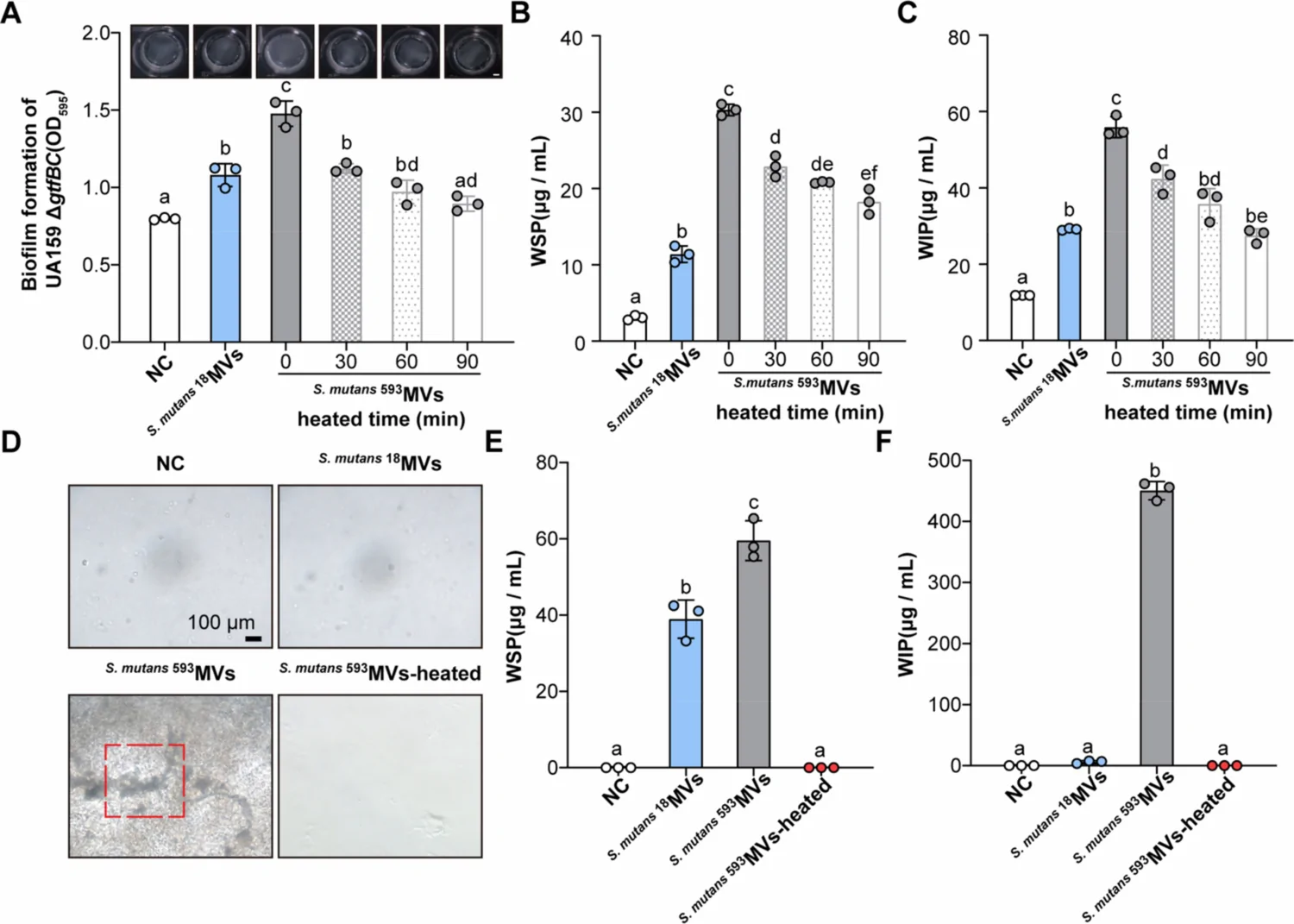
Effects of protein composition on MV function. (A, B, and C) Effect of ^*S. mutans* 18^MVs and heat inactivation (0, 30, 60, and 90 min at 70 °C) of ^*S. mutans* 593^MVs on UA159 Δ*gtfBC* biofilm formation on culture plates detected by crystal violet staining. Representative images of biofilms (upper panel; scale bar = 100 μm) and quantitative analysis of crystal violet staining (OD_595_) (lower panel) are shown (A). Quantification of WSP (B) and WIP (C) in the corresponding biofilms shown in (A) using the anthrone–sulfuric acid assay. NC, UA159 Δ*gtfBC* treated with PBS. (D) Representative images of extracellular polysaccharide aggregates formed by MVs incubated in a cell-free system containing 1% sucrose (BHIS) for 24 h. The boxed region depicts the flocculent polysaccharide aggregates generated by untreated ^*S. mutans* 593^MVs, which were absent after heat inactivation. Scale bar = 100 μm. (E, F) Quantification of WSP (E) and WIP (F) synthesized in the cell-free system shown in (D). NC, PBS with BHIS; ^*S. mutans* 18^MVs, *S. mutans* 18-derived MVs with BHIS; ^*S. mutans* 593^MVs, *S. mutans* 593-derived MVs with BHIS; ^*S. mutans* 593^MVs-heated, *S. mutans* 593-derived MVs heat-treated at 70 °C for 30 min before incubation. Data are presented as the mean ± SD from three independent biological replicates (*n* = 3). Different letters indicate significant differences (*P* < 0.05).

To determine whether MVs possess intrinsic catalytic activity independent of the biofilm microenvironment, the vesicles were incubated for 24 h in a cell-free reaction system containing 1% sucrose. Untreated ^*S. mutans* 593^MVs generated abundant, macroscopically visible flocculent aggregates, whereas no apparent polymer formation was observed after heat inactivation (Figure 7D; *P* < 0.05). Consistently, anthrone–sulfuric acid assays showed that active ^*S. mutans* 593^MVs produced substantially greater amounts of both WSP and WIP than heat-inactivated MVs and the other experimental groups. Polysaccharide production was markedly reduced following heat treatment (Figure 7E, F; *P* < 0.05). These findings suggest that MV preparations possess polysaccharide synthesis activity independent of viable bacterial cells.

## Discussion

The results of this study suggest that MVs secreted by *S. mutans* strains with different cariogenic potentials vary significantly in abundance, particle size, function, and protein cargo composition. MVs from highly cariogenic strains were more abundant and larger in size and were enriched in multiple virulence-associated proteins, thereby facilitating the recovery of the cariogenesis-deficient strain UA159 Δ*gtfBC* from defects in biofilm formation, extracellular matrix synthesis, and the establishment of an acidic microenvironment. These findings suggest that MVs may act as virulence factors of *S. mutans*, providing a theoretical basis for a deeper understanding of the pathogenic mechanism of *S. mutans*.

The colonization capacity of *S. mutans* is closely associated with its cariogenicity, and the two are thought to be causally related [53]. Colonization is a prerequisite for caries development. In our rat caries model, weakly cariogenic strains resulted in lower viable bacterial counts on the tooth surface (Figure 1C), which is consistent with the trends observed in our previous in vitro studies [34,35]. This suggests that highly cariogenic strains may exert their cariogenic effects through stronger colonization ability. However, the present data cannot separate the effects of MVs from those of colonization fitness.

As vital extracellular mediators, MVs help *S. mutans* withstand external stressors and maintain bacterial viability. They promote bacterial adherence to and colonization of oral epithelial surfaces [54], increase biofilm formation [55], and facilitate immune evasion by suppressing macrophage phagocytosis [56]. On the basis of these bioactivities, we isolated and characterized MVs from our experimental strains. MVs were harvested from stationary-phase cultures, as vesicles recovered at this growth phase have been reported to be more abundant and more stable [57]. SEM revealed that MVs appeared to bud directly from the bacterial cell surface (Figure 2A). The isolated vesicles exhibited a typical lipid bilayer structure and averaged 100–150 nm in diameter, which is consistent with published morphological and size data for MVs from *S. mutans* and other Gram-positive bacteria (Figure 2B, C, and D) [57,58]. Integrated proteomic and subcellular localization analyses revealed that MV-associated proteins are derived predominantly from the cytoplasm and cytoplasmic membrane (Figure 5C), a distribution that mirrors reports for *Staphylococcus aureus* MVs [46]. Together, these morphological and proteomic features indicate that MVs are unlikely to be incidental byproducts of membrane remodeling; rather, they appear to be functionally specialized structures whose biogenesis and release are actively regulated by the bacteria.

To explore the link between vesicle output and cariogenicity, we quantified MV secretion across strains. Previous work has suggested an association between MV biogenesis and cariogenic phenotypes [31,32], which is consistent with our findings (Figure 2F and G). In Gram-positive bacteria, MV release is an active strategy for coping with environmental stress, as exposure to antibiotics [59], acid [60], or oxidative stress [61] can markedly upregulate MV production. We therefore hypothesize that the high MV output of strongly cariogenic strains may confer a more efficient collective stress response within microenvironments characterized by nutrient fluctuations or acid challenge.

The biogenesis mechanisms of MVs in Gram-positive bacteria remain incompletely understood, with proposed contributions from changes in intracellular turgor pressure [62], cell wall hydrolysis and remodeling [46], and transmembrane transport systems [63,64]. In *S. mutans*, MV biogenesis has been linked to autolysins (LytF [47] and AltA [61]) and ABC transport machinery [60,65]. Consistent with these observations, we observed that AltA was enriched in MVs from both weakly and highly cariogenic strains (Figure 5E, F), whereas ABC transport-related proteins were significantly enriched specifically in MVs from the weakly cariogenic strain (Figure 6B), suggesting that autolysins and transmembrane transport systems may both participate in MV release, potentially in a strain-dependent manner. Nevertheless, detailed mechanistic evidence remains limited and warrants further experimental validation [65].

In addition to differing secretion yields, MV size influences protein loading capacity [66]. We found that MVs from *S. mutans* 593 were 1.14-fold larger in diameter than those from *S. mutans* 18 (Figure 2C, D) and carried more protein (Figure 5B), which is in line with observations in *Helicobacter pylori* [66]. Accordingly, the structural and compositional heterogeneity of MVs may contribute to their diverse biological roles [61].

The regulatory role of MVs in biofilm formation is well documented [12,19,24,67]. In the present study, exogenous MVs significantly reversed the defects of UA159 Δ*gtfBC* in terms of exopolysaccharide synthesis, biofilm formation, viable cell maintenance within biofilms, and acid production relative to those of the untreated mutant, with the degree of rescue following a gradient that paralleled the cariogenicity of the donor strains (Figure 3A). This finding is consistent with the findings of Senpuku et al., who reported that UA159-derived MVs promoted biofilm formation by UA159 Δ*gtfBC* in a dose-dependent manner [28].

The functional heterogeneity of MVs is closely linked to differences in their cargo composition [68]. In our proteomic analysis, proteins associated with starch and sucrose metabolism were enriched in the *S. mutans* 593 MVs compared with the *S. mutans* 18 MVs (Figure 6B). This finding is consistent with reports that under acid or oxidative stress, MVs can preferentially enrich functional proteins that help parent bacteria adapt to environmental challenges and sustain virulence [60,61]. Whether this finding reflects selective sorting during MV biogenesis or simply mirrors differences in the corresponding cellular proteomes remains to be determined by directly comparing MV and whole-cell protein abundances. Following heat shock (Figure 7) or proteinase K treatment (Fig. S3), the ability of MVs to rescue biofilm formation and to metabolize sucrose into WIP was significantly reduced, suggesting that protein cargo plays an important role in mediating these functions. Furthermore, MVs themselves exhibited a certain degree of biological activity independent of viable bacterial cells, including the ability to metabolize sucrose on their own, which is consistent with the findings of Cao et al. [60]. Future studies could investigate the interactions between MVs treated with heat shock or proteinase K and other strains.

MVs not only influence exopolysaccharide matrix formation but also may participate in the construction of eDNA scaffolds. Compared with biofilms treated with MVs from the weakly cariogenic strain, biofilms treated with MVs from the highly cariogenic strain exhibited significantly greater eDNA accumulation (Figure 4E). We hypothesize that this difference may arise from two possible factors: MVs themselves may carry eDNA as an intrinsic cargo [25,69] and MVs may carry specific proteins that promote eDNA accumulation; for example, MVs from highly cariogenic strains were enriched with a putative competence-related protein encoded by *Smu82_05147*, which may be involved in the binding, uptake, and transport of exogenous DNA [70]. Nevertheless, further studies are needed to clarify the underlying reasons for this difference.

In addition to driving cariogenic phenotypes via their packaged functional components, MVs may also exert indirect effects by upregulating the transcription of acidogenesis-related genes, such as *ldh* (Figure 4G), in recipient bacteria. Existing reports indicate that MVs are capable of modulating the transcription of genes related to adhesion and quorum sensing [55].

This study has several limitations. First, the functional membrane vesicle preparation was not further purified; therefore, residual non-vesicular components may remain in the samples. Second, only three *S. mutans* strains were examined, which limits the generalizability of our findings; future studies should therefore include a broader range of clinical isolates and incorporate genetic background analyses. Third, this study used a single-species biofilm model, whereas the oral microbiota constitutes a complex ecosystem in which interspecies interactions and host factors may modulate MV functionality; multispecies and in vivo models are therefore warranted to validate the functional heterogeneity of MVs derived from different strains. Fourth, the MV-mediated functional rescue experiments lacked a wild-type UA159 control group, making it unclear whether MV treatment restored UA159 Δ*gtfBC* to WT levels or whether MVs exert broader biofilm-promoting effects beyond simple phenotypic rescue. Finally, this study lacked in vivo MV intervention experiments, and the causal relationship between MVs from highly cariogenic strains and caries development therefore warrants further investigation.

## Conclusion

This study demonstrated that *S. mutans* strains with different cariogenic phenotypes produce MVs that differ in abundance, physicochemical properties, protein composition, and in vitro biofilm-modulating activity, highlighting MV heterogeneity as a factor for further investigation in strain-specific cariogenicity.

## Supplementary Material

Revised_Supplementary_Materials_clean.docx

## Data Availability

All the raw and processed data supporting the findings of this work can be obtained from the corresponding author upon reasonable academic request. The raw proteomic data have been deposited into the ProteomeXchange Consortium via the PRIDE partner repository under the data set identifier PXD085379.
