# Supplementary material for: Membrane vesicle heterogeneity of Streptococcus mutans is associated with strain-specific cariogenicity
Source: J Oral Microbiol. 2026 Nov 12;18(1):2761687. doi: 10.1080/20002297.2026.2761687 (PMC13761053; doi:10.1080/20002297.2026.2761687)
Supplement: Supplementary file 1 [file ZJOM_A_2761687_SM3827.docx]

Supplementary Materials

**Membrane vesicle heterogeneity of *Streptococcus mutans* is associated with strain-specific cariogenicity**

Wang Han^a, b^, Wen Zhou^a, b^, Yousheng Zeng^a, b^, Yazhuo Li^a, b^, Yuze Yang^a, b^, Yu Zeng^a, b^, Guowu Gan^a, b*^, Shuai Chen^a, b*^, Xiaojing Huang^a, b*^

*^a^* *Clinical Research Center for Oral Tissue Deficiency Diseases of Fujian Province & Fujian Key Laboratory of Oral Diseases & Fujian Provincial Engineering Research Center of Oral Biomaterial, School and Hospital of Stomatology, Fujian Medical University, Fuzhou, China;*

*^b^* *Stomatological Key Laboratory of Fujian College and University & Institute of oral homeostasis & Research Center of Oral Tissue Engineering, Fujian Medical University, School and Hospital of Stomatology, Fujian Medical University, Fuzhou, China.*

**Supplementary Results**

**Table S1.** Bacterial strains used in this study

| Strain | Description | Source |
| --- | --- | --- |
| *S. mutans* 18 | Wild type | Laboratory stock |
| UA159 | Reference strain | Laboratory stock |
| *S. mutans* 593 | Wild type | Laboratory stock |
| UA159 Δ*gtfBC* | *gtfBC knockout mutant* | Presented by  Professor Li |

**Table S2**. Nucleotide sequences of primers were used in this study

| Primer | Sequence(5’-3’) | | References |
| --- | --- | --- | --- |
| *ldh*-F | AAAAACCAGGCGAAACTCGC | This study | |
| *ldh*-R | CTGAACGCGCATCAACATCA | This study | |
| *gtfB-F* | TGCCGCAGTCCCTTCTTATTC | This study | |
| *gtfB-R* | GCCATGTATTGCCCGTCATCT | This study | |
| *gtfC-F* | GTGCGCTACACCAATGACAGAG | This study | |
| *gtfC-R* | GCCTACTGGAACCCAAACACCTA | This study | |
| *16S*-F | AGCGTTGTCCGGATTTATTG | This study | |
| *16S*-R | CTACGCATTTCACCGCTACA | This study | |

**Table S3**. The upregulated proteins in *^S. mutans^* ^593^MVs / *^S. mutans^* ^18^MVs (log_2_ (fold change) > 1 and *P*-adjust < 0.05)

| **UniProt** | **Gene name** | **log2FoldChange** | ***P-*value** | ***P-*adjust** |
| --- | --- | --- | --- | --- |
| A0A829BVF3 | *SMU82_05147* | 3.34E+00 | 4.56E-04 | 1.93E-02 |
| P08987 | *gtfB* | 2.03E+00 | 7.18E-03 | 5.27E-02 |
| P13470 | *gtfC* | 1.96E+00 | 1.28E-02 | 7.25E-02 |
| Q8DVU8 | *SMU_367* | 1.57E+00 | 3.51E-02 | 1.31E-01 |

**Table S4**. The downregulated proteins in *^S. mutans^* ^593^MVs / *^S. mutans^* ^18^MVs (log_2_ (fold change) < -1 and *P*-adjust < 0.05)

| **UniProt** | **Gene name** | **log2FoldChange** | ***P-*value** | ***P-*adjust** |
| --- | --- | --- | --- | --- |
| Q8DTB0 | *SMU_1447c* | -4.32E+00 | 1.64E-05 | 7.62E-03 |
| A0A829BN51 | *SMU82_09372* | -4.89E+00 | 5.26E-05 | 9.31E-03 |
| Q8DV98 | *folT* | -4.56E+00 | 6.49E-05 | 9.31E-03 |
| A0A829BTN1 | *SMU82_01776* | -3.75E+00 | 8.01E-05 | 9.31E-03 |
| A0A829BTX4 | *SMU82_06424* | -3.54E+00 | 1.27E-04 | 1.18E-02 |
| I6L927 | *atmB* | -4.23E+00 | 1.90E-04 | 1.26E-02 |
| Q8DUS9 | *SMU_817* | -3.59E+00 | 1.76E-04 | 1.26E-02 |
| A0A829BPR0 | *SMU82_07771* | -2.46E+00 | 2.21E-04 | 1.28E-02 |
| Q00749 | *msmE* | -3.37E+00 | 2.52E-04 | 1.30E-02 |
| A0A829BQ87 | *SMU82_01050* | -4.00E+00 | 3.05E-04 | 1.42E-02 |
| A0AAX1K2I9 | *IGS65_000420* | -3.76E+00 | 6.50E-04 | 2.03E-02 |
| A0A829BJ71 | *SMU82_05522* | -2.96E+00 | 6.55E-04 | 2.03E-02 |
| A0A829BUN1 | *ftsE* | -2.16E+00 | 5.45E-04 | 2.03E-02 |
| P96993 | *galK* | -1.95E+00 | 5.73E-04 | 2.03E-02 |
| A0A829BLY8 | *SMU82_06679* | -3.14E+00 | 7.41E-04 | 2.15E-02 |
| Q8DTY4 | *SMU_1177c* | -2.93E+00 | 7.95E-04 | 2.17E-02 |
| Q8DSQ4 | *SMU_1719c* | -2.61E+00 | 8.94E-04 | 2.31E-02 |
| A0A829BLT7 | *SMU82_05989* | -2.36E+00 | 1.02E-03 | 2.51E-02 |
| A0AAX1K2E1 | *IGS65_008915* | -3.17E+00 | 1.45E-03 | 3.22E-02 |
| A0A829BPU6 | *SMU82_06694* | -2.67E+00 | 1.45E-03 | 3.22E-02 |
| A0A829BQB5 | *SMU82_06619* | -2.64E+00 | 1.93E-03 | 3.51E-02 |
| A0A829BKK4 | *SMU82_05772* | -2.41E+00 | 1.96E-03 | 3.51E-02 |
| A0A829BTZ4 | *SMU82_07776* | -1.96E+00 | 1.84E-03 | 3.51E-02 |
| Q8DV07 | *murM* | -1.95E+00 | 1.87E-03 | 3.51E-02 |
| Q8DSX2 | *glmU* | -1.84E+00 | 1.68E-03 | 3.51E-02 |
| A0A829BKE3 | *rqcH* | -1.96E+00 | 2.36E-03 | 4.07E-02 |
| Q8CVC6 | *prsA* | -3.20E+00 | 3.15E-03 | 4.30E-02 |
| A0AAX1K288 | *IGS65_007530* | -2.76E+00 | 3.27E-03 | 4.30E-02 |
| A0A829BQ61 | *SMU82_06244* | -2.68E+00 | 3.81E-03 | 4.30E-02 |
| Q8DU40 | *SMU_1116c* | -2.49E+00 | 4.16E-03 | 4.30E-02 |
| A0A829BNL1 | *yajC* | -2.36E+00 | 3.81E-03 | 4.30E-02 |
| Q9KIJ3 | *sloC* | -2.34E+00 | 3.86E-03 | 4.30E-02 |
| A0A829BSI2 | *mltG* | -2.34E+00 | 2.79E-03 | 4.30E-02 |
| A0A829BYG0 | *SMU82_01300* | -2.24E+00 | 3.94E-03 | 4.30E-02 |
| A0A829BEZ3 | *SMU82_09387* | -2.04E+00 | 3.14E-03 | 4.30E-02 |
| A0AAX1K0V7 | *pbp2x* | -1.97E+00 | 3.58E-03 | 4.30E-02 |
| A0A829BH82 | *SMU82_08345* | -1.74E+00 | 3.76E-03 | 4.30E-02 |
| Q8DSD1 | *mutS2* | -1.58E+00 | 2.72E-03 | 4.30E-02 |
| Q8DSF0 | *secA* | -1.44E+00 | 4.04E-03 | 4.30E-02 |
| P72481 | *uvrA* | -1.42E+00 | 3.45E-03 | 4.30E-02 |
| Q8DS19 | *rplV* | -1.40E+00 | 4.00E-03 | 4.30E-02 |
| Q8DT55 | *phsG* | -1.39E+00 | 3.38E-03 | 4.30E-02 |
| Q8DT12 | *thrS* | -1.27E+00 | 4.11E-03 | 4.30E-02 |
| Q8CWX0 | *glgA* | -2.05E+00 | 4.48E-03 | 4.43E-02 |
| Q8DUR1 | *thiI* | -1.42E+00 | 4.40E-03 | 4.43E-02 |
| P12655 | *scrA* | -2.22E+00 | 4.87E-03 | 4.44E-02 |
| A0A829BT22 | *SMU82_02861* | -1.97E+00 | 4.74E-03 | 4.44E-02 |
| Q8DS22 | *rpsQ* | -1.68E+00 | 4.83E-03 | 4.44E-02 |
| P27756 | *aga* | -1.39E+00 | 4.68E-03 | 4.44E-02 |
| Q8DVX6 | *SMU_333* | -2.24E+00 | 4.96E-03 | 4.44E-02 |
| Q8DT54 | *glgD* | -1.82E+00 | 5.56E-03 | 4.88E-02 |
| Q8DV10 | *ppc* | -1.64E+00 | 5.74E-03 | 4.94E-02 |

**Figure S1**. Bacterial identification

**(A)** Phylogenetic analysis. **(B)** Gram stain. **(C)** Growth curve. **(D)** CFUs of bacterial suspension at OD_600_ = 0.5. Data are presented as the mean ± SD from three independent biological replicates (n = 3). **** *P* < 0.0001.

**Figure S2**. Identification of UA159 Δ*gtfBC*

**(A)** Gram stain. **(B)** RT‒qPCR detection of *gtfB* and *gtfC* in planktonic UA159 Δ*gtfBC*. **(C)** Growth curve. Data are presented as the mean ± SD from three independent biological replicates (n = 3). *****P* < 0.0001

**Figure S3.** Phenotypic complementation and protein cargo effects of MVs derived from strains with different cariogenic potentials at an equivalent particle concentration (2 × 10^7^ particles/mL).

**(A)** Representative images of crystal violet-stained biofilms in each treatment group. Scale bar = 100 μm. **(B)** Quantitative analysis of biofilm biomass. **(C)** Quantification of water-insoluble polysaccharide (WIP) using the anthrone-sulfuric acid assay. **(D)** Biofilm acidogenicity was assessed by an acid production assay. NC, negative control (UA159 Δ*gtfBC* with PBS); PK, proteinase K (UA159 Δ*gtfBC* treated with proteinase K); *^S. mutans^* ^18^MVs, UA159 Δ*gtfBC* treated with MVs derived from *S. mutans* 18; ^UA159^MVs, UA159 Δ*gtfBC* treated with MVs derived from UA159; *^S. mutans^* ^593^MVs, UA159 Δ*gtfBC* treated with MVs derived from *S. mutans* 593; MVs + PK, UA159 Δ*gtfBC* treated with MVs from each group after preincubation with proteinase K. Data are presented as the mean ± SD from three independent biological replicates (n = 3). Different letters indicate statistically significant differences (*P* < 0.05).

**Figure S4**. Protein Composition and Functions of MVs Containing Different Proteins **(A)** SDS‒PAGE of MVs and cells. Each lane contained 10 μg of protein. **(B)** Upper: Representative images of crystal violet-stained biofilms in each treatment group. Scale bar = 100 μm. Lower: Quantitative analysis of biofilm biomass. NC, negative control (UA159 Δ*gtfBC* with PBS); *^S. mutans^* ^18^MVs, UA159 Δ*gtfBC* treated with MVs derived from *S. mutans* 18; ^UA159^MVs, UA159 Δ*gtfBC* treated with MVs derived from UA159; *^S. mutans^* ^593^MVs, UA159 Δ*gtfBC* treated with MVs derived from *S. mutans* 593; ^UA159 Δ^*^gtfBC^*MVs, UA159 Δ*gtfBC* treated with MVs derived from UA159 Δ*gtfBC.* Data are presented as the mean ± SD from three independent biological replicates (n = 3). Different letters indicate statistically significant differences (*P* < 0.05).
